# Supplementary material for: CBGTPy: An extensible cortico-basal ganglia-thalamic framework for modeling biological decision making
Source: PLoS One. 2025 Jan 14;20(1):e0310367. doi: 10.1371/journal.pone.0310367 (PMC11731724; doi:10.1371/journal.pone.0310367)
Supplement: S1 Appendix — (PDF) [file pone.0310367.s001.pdf]

S1 Appendix CBGT network

S1.1 Overview of CBGT pathways

While many of the circuit-level details of the basal ganglia (BG) pathways are complex, with new cell types and connections being discovered with increased frequency as our biological tools improve, the consensus conceptualization of the canonical BG circuits has long remained stable [1–3]. Much of the theoretical work on this topic relates to a profoundly influential framework for representing the passage of top-down signals through the BG, which describes the network as a collection of feed-forward pathways, starting from a cortical input source and flowing to output units that project to certain thalamic nuclei and other subcortical targets [4–8] (Fig 1 in the manuscript). In the *direct pathway*, cortical inputs drive a subpopulation of spiny projection neurons (dSPNs) in the striatum. These dSPNs send inhibitory projections directly to basal ganglia output units, which we refer to as the globus pallidus internal segment (GPi), but which can be tuned to represent other outputs such as the substantia nigra pars reticulata (SNr) to suit a user's interests. As these output units are comprised of GABAergic neurons that are suppressed by inhibition from the dSPNs, the traditional view posits that the direct pathway may act to facilitate action selection by disinhibiting populations downstream from the BG.

The traditional *indirect pathway* starts with cortical inputs to a second striatal subpopulation of spiny projection neurons (iSPNs). Like dSPNs, iSPNs send inhibition to the globus pallidus, specifically its external segment (GPe). Unlike the GPi, however, the GPe is not itself an output unit. GABAergic GPe efferents project to the GPi/SNr, to another region called the subthalamic nucleus (STN), and back to the striatum itself. The GPe feedback signals to the striatum, part of what is called the pallidostriatal circuit, rely both on prototypical (GPeP) and arkypallidal (GPeA) GPe cells [9] and are not considered part of the indirect pathway [10]. The STN relays signals to the GPi/SNr, but via glutamatergic rather than GABAergic synapses. Although complex in its structural organization, the "indirect" pathway framework produces a simple prediction: the net effect of cortical activation of iSPNs will be to inhibit GPe neurons. As a result, GPi/SNr neurons are directly disinhibited, and STN neurons are also disinhibited, which yields a surge in excitation to GPi/SNr that further enhances their firing and hence the suppression of downstream targets.

A key piece for adapting this framework to action selection is the concept of action channels. These channels represent putative parallel pathways, each responsible for whether a specific action (perhaps a specific muscle contraction or limb movement, perhaps the performance of a more complete action comprising multiple movements) is implemented or suppressed [6]. Thus, the framework conceptualizes the basal ganglia as a collection of independent action channels [6], with evidence accumulation or other processing occurring in parallel across channels, until this competition results in victory for one action, while the others are suppressed (i.e., winner-take-all selection [11]). Outside of these canonical pathways, however, a third *hyperdirect pathway* stands ready to relay excitation from cortex directly to the STN, providing a proposed mechanism for reactive stopping that can abruptly interrupt or block all actions [7].

Beyond action selection, the CBGT pathways are also notable for their critical role in learning. Nigrostriatal pathways send dopaminergic projections directly to the striatal SPNs [12], where the dSPNs and iSPNs predominantly express D1 and D2 dopamine receptors, respectively [13]. Phasic dopamine signals differentially modulate D1 and D2 pathways in response to post-action feedback [14], sculpting corticostriatal synapses over time to effectively promote or inhibit actions in order to maximize future returns. The critical learning signal arises from a discrepancy between a received reward and the expected reward, known as the reward prediction error (RPE), which appears

to be encoded in the firing of dopaminergic neurons in the substantia nigra pars compacta (SNc) [15]. Corticostriatal neural plasticity depends on the levels and timing of dopamine signals, which leads to computational models of reinforcement learning that integrate the RPE concept [14, 16–18]; such a model has even been used to suggest the computational underpinning for the temporal profile of the dopamine signal [19].

Of course, the reality of the CBGT pathways is more complicated than would expected from the simple canonical model. For example, from the onset of a decision process to the execution of an action, dSPN and iSPN activity has been observed to co-vary, contrasting with the idea that the two subpopulations activate at different times as simple "go" or "no-go" switches, respectively [20–23]. In addition, activity of these pathways has been found to impact the kinematics of a movement that is made, rather than or in addition to which action is chosen [24–28]. Continuous stimulation of the STN with deep brain stimulation in individuals with parkinsonism has been shown to fundamentally change BG output [29], impacting impulsiveness without compromising the selection of learned actions. Finally, the idea that hyperdirect pathway activation of the STN acts as a brake that can prevent planned actions via direct activation of the GPi has come into doubt as new cell types (e.g., arkypallidal cells in the GPe) and new connections (e.g., GPe arkypallidal outputs and thalamic projections to the striatum), have been recognized as playing critical roles in stopping [10, 30–32].

S1.2 CBGT model details

The total number of neurons per population is provided in Table S1_1.

Population	CxI	Cx	$dSPN$	$iSPN$	FSI	GPe_A	GPe_P	GPi	STN	Th
Number of neurons	186	204	75	75	75	190	560	75	750	75

Table S1_1. Number of neurons considered in each population. When no distinction between GPe_A and GPe_P is considered, the total number of neurons at GPe is the sum of arkypallidals and prototypicals (750).

As in previous works [33–35], the activity of each neuron is described by the integrate-and-fire-or-burst model [36], with equations given by

$$
\begin{aligned}
C\frac{dV}{dt} &= -g_L(V(t)-V_L) - g_T h(t) H(V(t)-V_h)(V(t)-V_T) - I_{syn}(t) + I_{ext}(t),\\
\frac{dh}{dt} &= \begin{cases} -h(t)/\tau_h^- & \text{when } V \geq V_h,\\ -(1-h(t))/\tau_h^+ & \text{when } V < V_h,\end{cases}
\end{aligned} \quad (1)
$$

where $V(t)$ denotes the activity of the membrane potential at time t. The equation describing the evolution of the membrane potential (dV/dt) contains the leak current, with constant conductance g_L and reversal potential V_L; the low-threshold Ca^{2+} current, with constant conductance g_T, gating variable $h(t)$, and reversal potential V_T; the synaptic current $I_{syn(t)}$; and, finally, the external current $I_{ext}(t)$. Parameter C stands for the capacitance of the membrane potential. The evolution (dh/dt) of the gating variable $h(t)$ changes according to the relation of V to a constant voltage threshold for burst activation, V_h, where τ_h^+ and τ_h^- represent, respectively, the decay time constant when the membrane potential is below or above V_h . Finally, $H(\cdot)$ represents the Heaviside step function. As with all integrate-and-fire models, a reset condition is added to the model to control the spike generation such that if $V(t)$ crosses a certain threshold value V_{th}, then the membrane potential is reset to ta hyperpolarized membrane potential V_{re}, simulating the spike onset time. That is, if $V(t^-) > V_{th}$, then $V(t^+) = V_{re}$ and a spike has been made by the specific neuron. Parameters of the neuronal model are provided in Table S1_2.

Population	$C\,(nF)$	$g_L\,(nS)$	$g_T\,(nS)$	$V_L\,(mV)$	$V_h\,(mV)$	$V_T\,(mV)$	$V_{th}\,(mV)$	$V_{re}\,(mV)$	$\tau_h^-\,(ms)$	$\tau_h^+\,(ms)$
CxI	1	1/10	0	−55	−60	120	−50	−55		
Cx	1	1/20	0	−55	−60	120	−50	−55	20	100
$dSPN$	1	1/20	0	−55	−60	120	−50	−55	20	100
$iSPN$	1	1/20	0	−55	−60	120	−50	−55	20	100
FSI	1	1/10	0	−55	−60	120	−50	−55	20	100
$^*GPe$	1	1/20	0.06	−55	−60	120	−50	−55	20	100
GPe_A	1	1/20	0.06	−55	−60	120	−50	−55	20	100
GPe_P	1	1/20	0.06	−55	−60	120	−50	−55	20	100
GPi	1	1/20	0	−55	−60	120	−50	−55	20	100
STN	1	1/20	0.06	−55	−60	120	−50	−55	20	100
Th	1	1/27.78	0	−55	−60	120	−50	−55	20	100

Table S1_2. Neuronal parameters. Parameters used in the integrate-and-fire-or-burst model (see equation (1)) where C is the membrane capacitance and coincide with the inverse of the membrane time constant, g_L is the leak conductance, g_T is the low threshold Ca^{2+} maximal conductance, V_L is the leak reversal potential, V_h is the threshold potential for the burst activation, V_T is the low threshold Ca^{2+} reversal potential, τ_h^- is the burst duration, and τ_h^+ is the hyperpolarization duration. * Values in this row are the ones used when no intrinsic separation of neurons is considered.

All neurons in the network communicate through the simulated release of neurotransmitters across synapses. When action potentials arrive at postsynaptic neurons, the activity of each neuron's AMPA, GABA, and NMDA receptors increases according to the synaptic weight and neurotransmitter type. The activation of these receptors induces cellular currents which, in turn, drive future action potentials. The synaptic current $I_{syn}(t)$ is therefore modeled as

$$\begin{aligned} I_{syn}(t) = \quad & g_{AMPA}s_{AMPA}(t)(V(t) - V_E) + \frac{g_{NMDA}s_{NMDA}(t)}{,} 1 + e^{-0.062V(t)/3.57}(V(t) - V_E) \\ & + g_{GABA}s_{GABA}(t)(V(t) - V_I), \end{aligned}$$

where g_x, for $x \in \{AMPA,\ NMDA,\ GABA\}$, stands for the maximal conductance in each channel; V_E and V_I are the excitatory and inhibitory reversal potentials, respectively; and finally, the variable $s_x(t)$, for $x \in \{AMPA,\ NMDA,\ GABA\}$, corresponds to the fraction of open channels of each type. The latter variables evolve according to the differential equations

$$\begin{aligned} \frac{ds_{AMPA}}{dt} &= \textstyle\sum_j \delta(t - t_j) - \frac{s_{AMPA}}{\tau_{AMPA}}, \\ \frac{ds_{NMDA}}{dt} &= \alpha(1 - s_{NMDA}) \textstyle\sum_j \delta(t - t_j) - \frac{s_{NMDA}}{\tau_{NMDA}}, \\ \frac{ds_{GABA}}{dt} &= \textstyle\sum_j \delta(t - t_j) - \frac{s_{GABA}}{\tau_{GABA}} \end{aligned}$$

where t_j stands for the $j-$th spike onset time; α is a constant rate; τ_x, for $x \in \{AMPA,\ NMDA,\ GABA\}$, is the decay time constant of the corresponding s_x. The term $(1 - s_{NMDA})$ has been designed in order to prevent s_{NMDA} from exceeding the value of 1. Finally, $\delta(\cdot)$ stands for the Dirac delta function.

Individual neurons within the same population are connected to each other with a population-specific probability (p_x) and connection strength (weights, w_x), such that the maximal conductance for a specific receptor x, for $x \in \{AMPA,\ NMDA,\ GABA\}$, is given by $g_x = p_x w_x$. Connections between populations similarly are characterized by probabilities and strengths. These connections are depicted with arrows in Fig 1 in the manuscript. The direct (green connections), indirect (blue connections), and pallidostriatal (yellow connections) pathways are present. Depending on the type of receptors, these connections can be inhibitory (arrows ending in a circle) or excitatory

(arrows ending in a triangle). Parameters used for the connectivity are provided in Table S1_3.

All populations have an external current I_{ext} to tune their baseline firing rate, given by

$$I_{ext}(t) = S_{ext,AMPA}(V(t) - V_E) + S_{ext,GABA}(V(t) - V_I)$$

where $S_{ext,x}$ for $x \in \{AMPA,\, GABA\}$ is a mean-reverting random walk derived from the stochastic differential equation

$$dS_{ext,x} = \frac{(\mu_{ext,x} - S_{ext,x})}{\tau_x} dt + \sigma_{ext,x}\sqrt{\frac{2}{\tau_x}} dW_t.$$

Here, W_t is a Wiener process, τ_x is the time decay of the external current, and $\mu_{ext,x}$ and $\sigma_{ext,x}$ are computed as

$$\mu_{ext,x} = 0.001 E_{ext,x} f_{ext,x} N_{ext,x} \tau_x,$$
$$\sigma_{ext,x} = E_{ext,x}\sqrt{0.0005 f_{ext,x} N_{ext,x} \tau_x}.$$

The parameter $f_{ext,x}$ is the external input frequency, $E_{ext,x}$ is the mean efficacy of the external connections, $N_{ext,x}$ is the number of connections, and τ_x the time decay constant. Values of all of these parameters are specified in Tables S1_3 and S1_4. Connections were adjusted to reflect empirical knowledge about local and distal connectivity associated with different populations (see [33, 34, 37, 38] and the specific connection references in Table S1_3), as well as resting and task-related firing patterns (see Table S1_5 and [35]). We note that our connection probabilities are generally high (unlike some experimental work such as [39]) due to the fact that we simulate a small number of action channels [40]. In addition, the SPN outputs have been scaled to reflect the fact that the SPN population in our model is relatively small.

all pathways network				direct/indirect pathways network			
Connected populations	Receptor type	Connection Probability	Connection strength	Connected populations	Receptor type	Connection Probability	Connection strength
$CxI - CxI$	GABA	1	1.075	$CxI - CxI$	GABA	1.0	1.075
$CxI - Cx$	GABA	0.5	1.05	$CxI - Cx$	GABA	0.5	1.05
$Cx - Cx$	AMPA	0.13	0.0127	$Cx - Cx$	AMPA	0.13	0.0127
	NMDA	0.13	0.1		NMDA	0.13	0.08
$Cx - CxI$	AMPA	0.0725	0.113	$Cx - CxI$	AMPA	0.0725	0.113
	NMDA	0.0725	0.525		NMDA	0.0725	0.525
$Cx - dSPN$	AMPA	1	0.022	$Cx - dSPN$	AMPA	1.0	0.015
	NMDA	1	0.03,		NMDA	1.0	0.02
$Cx - iSPN$	AMPA	1	0.022	$Cx - iSPN$	AMPA	1.0	0.015
	NMDA	1	0.028		NMDA	1.0	0.02
$Cx - FSI$	AMPA	1	0.085	$Cx - FSI$	AMPA	1.0	0.19
$Cx - Th$	AMPA	1	0.025	$Cx - Th$	AMPA	1.0	0.025
	NMDA	1	0.029		NMDA	1.0	0.029
$dSPN - dSPN$	GABA	0.45	0.28	$dSPN - dSPN$	GABA	0.45	0.28
$dSPN - iSPN$	GABA	0.45	0.28	$dSPN - iSPN$	GABA	0.45	0.28
$dSPN - GPi$	GABA	1	1.8	$dSPN - GPi$	GABA	1.0	2.09
$dSPN - GPe_A$ [41–43]	GABA	0.4	0.054				
$iSPN - iSPN$	GABA	0.45	0.28	$iSPN - iSPN$	GABA	0.45	0.28
$iSPN - dSPN$	GABA	0.5	0.28	$iSPN - dSPN$	GABA	0.5	0.28
$iSPN - GPe_A$ [10, 41–43]	GABA	0.4	0.61	$iSPN - GPe$ [44, 45]	GABA	1.0	4.07
$iSPN - GPe_P$ [10, 41, 43]	GABA	1	4.07				
$FSI - FSI$	GABA	1	2.7	$FSI - FSI$	GABA	1.0	3.25833
$FSI - dSPN$ [46]	GABA	1	1.25	$FSI - dSPN$	GABA	1.0	1.2
$FSI - iSPN$ [46]	GABA	1	1.15	$FSI - iSPN$	GABA	1.0	1.1
$GPe_A - GPe_A$	GABA	0.4	0.15				
$GPe_A - iSPN$ [9, 43, 47–49]	GABA	0.4	0.12				
$GPe_A - dSPN$ [9, 43, 47–49]	GABA	0.4	0.32				
$GPe_A - FSI$ [9, 41–43, 47–49]	GABA	0.4	0.01				
$GPe_P - GPe_P$	GABA	0.4	0.45	$GPe - GPe$	GABA	0.0667	1.75
$GPe_P - GPe_A$ [10, 43, 50–52]	GABA	0.5	0.3				
$GPe_P - STN$ [41, 47, 48, 53, 54]	GABA	0.1	0.37	$GPe - STN$ [55]	GABA	0.0667	0.35
$GPe_P - GPi$ [41, 47, 48, 53, 54]	GABA	1	0.058	$GPe - GPi$	GABA	1.0	0.058
$GPe_P - FSI$ [9, 42, 47, 48, 54]	GABA	0.4	0.1				
$STN - GPe_P$ [10, 43, 56]	AMPA	0.161666	0.10	$STN - GPe$ [55]	AMPA	0.161666	0.07
	NMDA	0.161666	1.51		NMDA	0.161666	1.51
$STN - GPe_A$ [10, 43, 56]	AMPA	0.161666	0.026				
	NMDA	0.161666	0.075				
$STN - GPi$	AMPA	1	0.0325	$STN - GPi$	AMPA	1.0	0.038
$GPi - Th$	GABA	1	0.3315	$GPi - Th$	GABA	1.0	0.3315
$Th - dSPN$	AMPA	1	0.3285	$Th - dSPN$	AMPA	1.0	0.3825
$Th - iSPN$	AMPA	1	0.3285	$Th - iSPN$	AMPA	1.0	0.3825
$Th - FSI$	AMPA	0.8334	0.1	$Th - FSI$	AMPA	0.8334	0.1
$Th - Cx$	NMDA	0.8334	0.03	$Th - Cx$	AMPA	0.8334	0.03
$Th - CxI$	NMDA	0.8334	0.015	$Th - CxI$	AMPA	0.8334	0.015

Table S1_3. CBGT connectivity parameters. Two blocks of 4 columns each are depicted. The first block contains information regarding the network when the 4 different pathways are simulated (see Fig 1 in the manuscript), while the second block contains information regarding the network when only direct/indirect pathways are simulated. Columns in each block describe the parameters used to compute, in each population (1st columns), the maximal conductances g_x, for $x \in \{AMPA, NMDA, GABA\}$ (2nd column), which is the product of the probability of connectivity (3rd column) times the strength of connection (4th column). The rest of parameters are common such that $\tau_{AMPA} = 2\,ms$, $\tau_{NMDA} = 100\,ms$, $\tau_{GABA} = 5\,ms$, $V_E = 0\,mV$, $V_I = -70\,mV$, and $\alpha = 0.6332$.

all pathways network					direct/indirect pathways network				
Population	Receptor	External input frequency (spikes/s)	External connection efficacy (nS)	External connections number	Population	Receptor	External input frequency (spikes/s)	External connection efficacy (nS)	External connections number
CxI	AMPA	3.7	1.2	640	CxI	AMPA	3.7	1.2	640
Cx	AMPA	2.5	2.0	800	Cx	AMPA	2.3	2.0	800
$dSPN$	AMPA	1.3	4.0	800	$dSPN$	AMPA	1.3	4.0	800
$iSPN$	AMPA	1.3	4.0	800	$iSPN$	AMPA	1.3	4.0	800
FSI	AMPA	4.8	1.55	800	FSI	AMPA	3.6	1.55	800
GPe_A	GABA	2.0	2.0	2000					
	AMPA	2.5	2.0	800					
GPe_P	GABA	2.0	2.0	2000	$^*GPe$	GABA	2.0	2.0	2000
	AMPA	4.0	2.0	800		AMPA	4.0	2.0	800
GPi	AMPA	0.84	5.9	800	GPi	AMPA	0.8	5.9	800
STN	AMPA	4.45	1.65	800	STN	AMPA	4.45	1.65	800
Th	AMPA	2.2	2.5	800	Th	AMPA	2.2	2.5	800

Table S1_4. External current parameters. Parameters used to describe the external current (I_{ext}) arriving at the different populations of the CBGT network. From the third column to the last, we specify the different parameters used to describe the external current impinging in each population specified in column 1 and for the specific type of receptors. A non described receptor type means that the parameters are considered to be zero. The time decay constant τ is the same for all populations and only depends on the type of receptor being $\tau = 2\,ms$ if the receptor type is AMPA and $\tau = 5\,ms$ if it is GABA. * Values in this row are the ones used when no intrinsic separation of neurons is considered.

Population	baseline FR range (Hz)	full FR range (Hz)	References
dSPN	$[0, 5]$	$[0, 35]$	[57–60]
iSPN	$[0, 5]$	$[0, 35]$	[57–60]
GPe	$[40, 90]$	$[40, 150]$	[61–63]
GPi	$[40, 90]$	$[40, 150]$	[63]
STN	$[10, 35]$	$[10, 55]$	[61–63]
Th	$[5, 20]$	$[5, 85]$	[64]
Cx		$[0, 100]$	[65]
FSI	$[5, 40]$	$[5, 70]$	[65]

Table S1_5. Firing frequency ranges observed in different brain populations. The second column refers to the firing frequency ranges observed experimentally during baseline for each population set in the first column, whereas the third column refers to the ranges observed during decision tasks. In both cases, the ranges reflect experimental data from primates and rats (see references in the last column).

References

1. Chakravarthy VS, Joseph D, Bapi RS. What do the basal ganglia do? A modeling perspective. Biological cybernetics. 2010;103:237–253.
2. Dunovan K, Verstynen T. Believer-Skeptic meets actor-critic: Rethinking the role of basal ganglia pathways during decision-making and reinforcement learning. Frontiers in Neuroscience. 2016;10(MAR):1–15. doi:10.3389/fnins.2016.00106.
3. Bariselli S, Fobbs W, Creed M, Kravitz A. A competitive model for striatal action selection. Brain research. 2019;1713:70–79.
4. Alexander GE, DeLong MR, Strick PL. Parallel organization of functionally segregated circuits linking basal ganglia and cortex. Annual review of neuroscience. 1986;9(1):357–381.
5. Albin RL, Young AB, Penney JB. The functional anatomy of basal ganglia disorders. Trends in neurosciences. 1989;12(10):366–375.
6. Mink JW. The basal ganglia: focused selection and inhibition of competing motor programs. Progress in neurobiology. 1996;50(4):381–425.
7. Nambu A, Tokuno H, Takada M. Functional significance of the cortico–subthalamo–pallidal 'hyperdirect'pathway. Neuroscience research. 2002;43(2):111–117.
8. Kravitz AV, Tye LD, Kreitzer AC. Distinct roles for direct and indirect pathway striatal neurons in reinforcement. Nature neuroscience. 2012;15(6):816–818.
9. Mallet N, Micklem BR, Henny P, Brown MT, Williams C, Bolam JP, et al. Dichotomous organization of the external globus pallidus. Neuron. 2012;74(6):1075–1086.
10. Aristieta A, Barresi M, Lindi SA, Barriere G, Courtand G, de la Crompe B, et al. A disynaptic circuit in the globus pallidus controls locomotion inhibition. Current Biology. 2021;31(4):707–721.
11. Maass W. On the computational power of winner-take-all. Neural computation. 2000;12(11):2519–2535.
12. Hedreen JC, Delong MR. Organization of striatopallidal, striatonigral, and nigrostriatal projections in the macaque. Journal of Comparative Neurology. 1991;304(4):569–595.
13. Gerfen CR, Engber TM, Mahan LC, Susel Z, Chase TN, Monsma Jr FJ, et al. D1 and D2 dopamine receptor-regulated gene expression of striatonigral and striatopallidal neurons. Science. 1990;250(4986):1429–1432.
14. Gurney KN, Humphries MD, Redgrave P. A New Framework for Cortico-Striatal Plasticity: Behavioural Theory Meets In Vitro Data at the Reinforcement-Action Interface. PLoS Biology. 2015;13(1):e1002034. doi:10.1371/journal.pbio.1002034.
15. Schultz W. Predictive reward signal of dopamine neurons. Journal of Neurophysiology. 1998;80(1):1–27. doi:10.1152/jn.1998.80.1.1.
16. Bogacz R, Larsen T. Integration of reinforcement learning and optimal decision-making theories of the basal ganglia. Neural Computation. 2011;23(4):817–851. doi:10.1162/NECO_a_00103.

17. Frank MJ, Seeberger LC, O'reilly RC. By carrot or by stick: cognitive reinforcement learning in parkinsonism. Science (New York, NY). 2004;306(5703):1940–3. doi:10.1126/science.1102941.

18. Vich C, Dunovan K, Verstynen T, Rubin J. Corticostriatal synaptic weight evolution in a two-alternative forced choice task: a computational study. Communications in Nonlinear Science and Numerical Simulation. 2020;82:105048.

19. Morita K, Kato A. Striatal dopamine ramping may indicate flexible reinforcement learning with forgetting in the cortico-basal ganglia circuits. Frontiers in neural circuits. 2014;8(April):36. doi:10.3389/fncir.2014.00036.

20. Cui G, Jun SB, Jin X, Pham MD, Vogel SS, Lovinger DM, et al. Concurrent activation of striatal direct and indirect pathways during action initiation. Nature. 2013;494(7436):238.

21. Tecuapetla F, Matias S, Dugue GP, Mainen ZF, Costa RM. Balanced activity in basal ganglia projection pathways is critical for contraversive movements. Nature communications. 2014;5:4315.

22. Shin JH, Kim D, Jung MW. Differential coding of reward and movement information in the dorsomedial striatal direct and indirect pathways. Nature communications. 2018;9(1):1–14.

23. Parker JG, Marshall JD, Ahanonu B, Wu YW, Kim TH, Grewe BF, et al. Diametric neural ensemble dynamics in parkinsonian and dyskinetic states. Nature. 2018;557(7704):177–182.

24. Dudman JT, Krakauer JW. The basal ganglia: from motor commands to the control of vigor. Current opinion in neurobiology. 2016;37:158–166.

25. Turner RS, Desmurget M. Basal ganglia contributions to motor control: a vigorous tutor. Current opinion in neurobiology. 2010;20(6):704–716.

26. Rueda-Orozco PE, Robbe D. The striatum multiplexes contextual and kinematic information to constrain motor habits execution. Nature neuroscience. 2015;18(3):453–460.

27. Thura D, Cisek P. The basal ganglia do not select reach targets but control the urgency of commitment. Neuron. 2017;95(5):1160–1170.

28. Yttri EA, Dudman JT. Opponent and bidirectional control of movement velocity in the basal ganglia. Nature. 2016;533(7603):402–406.

29. Hashimoto T, Elder CM, Okun MS, Patrick SK, Vitek JL. Stimulation of the subthalamic nucleus changes the firing pattern of pallidal neurons. Journal of neuroscience. 2003;23(5):1916–1923.

30. Mallet N, Schmidt R, Leventhal D, Chen F, Amer N, Boraud T, et al. Arkypallidal cells send a stop signal to striatum. Neuron. 2016;89(2):308–316.

31. Schmidt R, Berke JD. A Pause-then-Cancel model of stopping: evidence from basal ganglia neurophysiology. Philosophical Transactions of the Royal Society B: Biological Sciences. 2017;372(1718):20160202.

32. Goenner L, Maith O, Koulouri I, Baladron J, Hamker FH. A spiking model of basal ganglia dynamics in stopping behavior supported by arkypallidal neurons. European Journal of Neuroscience. 2021;53(7):2296–2321. doi:10.1111/ejn.15082.

33. Wei W, Rubin JE, Wang XJ. Role of the indirect pathway of the basal ganglia in perceptual decision making. Journal of Neuroscience. 2015;35(9):4052–4064.

34. Dunovan K, Vich C, Clapp M, Verstynen T, Rubin J. Reward-driven changes in striatal pathway competition shape evidence evaluation in decision-making. PLoS computational biology. 2019;15(5):e1006998.

35. Vich C, Clapp M, Rubin JE, Verstynen T. Identifying control ensembles for information processing within the cortico-basal ganglia-thalamic circuit. PLOS Computational Biology. 2022;18(6):e1010255.

36. Smith GD, Cox CL, Sherman SM, Rinzel J. Fourier analysis of sinusoidally driven thalamocortical relay neurons and a minimal integrate-and-fire-or-burst model. J Neurophysiol. 2000;83(1):588–610.

37. Lo CC, Wang XJ. Cortico–basal ganglia circuit mechanism for a decision threshold in reaction time tasks. Nature neuroscience. 2006;9(7):956–963.

38. Kumar A, Cardanobile S, Rotter S, Aertsen A. The role of inhibition in generating and controlling Parkinson's disease oscillations in the basal ganglia. Frontiers in systems neuroscience. 2011;5:86.

39. Wilson CJ. Active decorrelation in the basal ganglia. Neuroscience. 2013;250:467–482.

40. Klaus A, Martins GJ, Paixao VB, Zhou P, Paninski L, Costa RM. The spatiotemporal organization of the striatum encodes action space. Neuron. 2017;95(5):1171–1180.

41. Bevan MD, Booth PA, Eaton SA, Bolam JP. Selective innervation of neostriatal interneurons by a subclass of neuron in the globus pallidus of the rat. Journal of Neuroscience. 1998;18(22):9438–9452.

42. Corbit VL, Whalen TC, Zitelli KT, Crilly SY, Rubin JE, Gittis AH. Pallidostriatal projections promote β oscillations in a dopamine-depleted biophysical network model. Journal of Neuroscience. 2016;36(20):5556–5571.

43. Ketzef M, Silberberg G. Differential synaptic input to external globus pallidus neuronal subpopulations in vivo. Neuron. 2021;109(3):516–529.

44. Fox C, Rafols J. The striatal efferents in the globus pallidus and in the substantia nigra. Research Publications-Association for Research in Nervous and Mental Disease. 1976;55:37–55.

45. Smith Y, Bevan M, Shink E, Bolam JP. Microcircuitry of the direct and indirect pathways of the basal ganglia. Neuroscience. 1998;86(2):353–387.

46. Gittis AH, Nelson AB, Thwin MT, Palop JJ, Kreitzer AC. Distinct roles of GABAergic interneurons in the regulation of striatal output pathways. Journal of Neuroscience. 2010;30(6):2223–2234.

47. Abdi A, Mallet N, Mohamed FY, Sharott A, Dodson PD, Nakamura KC, et al. Prototypic and arkypallidal neurons in the dopamine-intact external globus pallidus. Journal of Neuroscience. 2015;35(17):6667–6688.

48. Saunders A, Huang KW, Sabatini BL. Globus pallidus externus neurons expressing parvalbumin interconnect the subthalamic nucleus and striatal interneurons. PloS one. 2016;11(2):e0149798.

49. Hernández VM, Hegeman DJ, Cui Q, Kelver DA, Fiske MP, Glajch KE, et al. Parvalbumin+ neurons and Npas1+ neurons are distinct neuron classes in the mouse external globus pallidus. Journal of Neuroscience. 2015;35(34):11830–11847.

50. Nevado-Holgado AJ, Mallet N, Magill PJ, Bogacz R. Effective connectivity of the subthalamic nucleus - globus pallidus network during Parkinsonian oscillations. The Journal of physiology. 2014; p. 1–12. doi:10.1113/jphysiol.2013.259721.

51. Dodson PD, Larvin JT, Duffell JM, Garas FN, Doig NM, Kessaris N, et al. Distinct developmental origins manifest in the specialized encoding of movement by adult neurons of the external globus pallidus. Neuron. 2015;86(2):501–513.

52. Fujiyama F, Nakano T, Matsuda W, Furuta T, Udagawa J, Kaneko T. A single-neuron tracing study of arkypallidal and prototypic neurons in healthy rats. Brain Structure and Function. 2016;221:4733–4740.

53. Mastro KJ, Bouchard RS, Holt HA, Gittis AH. Transgenic mouse lines subdivide external segment of the globus pallidus (GPe) neurons and reveal distinct GPe output pathways. Journal of Neuroscience. 2014;34(6):2087–2099.

54. Glajch KE, Kelver DA, Hegeman DJ, Cui Q, Xenias HS, Augustine EC, et al. Npas1+ pallidal neurons target striatal projection neurons. Journal of Neuroscience. 2016;36(20):5472–5488.

55. Steiner LA, Tomás FJB, Planert H, Alle H, Vida I, Geiger JR. Connectivity and dynamics underlying synaptic control of the subthalamic nucleus. Journal of Neuroscience. 2019;39(13):2470–2481.

56. Pamukcu A, Cui Q, Xenias HS, Berceau BL, Augustine EC, Fan I, et al. Parvalbumin+ and Npas1+ pallidal neurons have distinct circuit topology and function. Journal of Neuroscience. 2020;40(41):7855–7876.

57. Kimura M. Behavioral modulation of sensory responses of primate putamen neurons. Brain research. 1992;578(1-2):204–214.

58. Aosaki T, Graybiel AM, Kimura M. Effect of the nigrostriatal dopamine system on acquired neural responses in the striatum of behaving monkeys. Science. 1994;265(5170):412–415.

59. Barnes TD, Kubota Y, Hu D, Jin DZ, Graybiel AM. Activity of striatal neurons reflects dynamic encoding and recoding of procedural memories. Nature. 2005;437(7062):1158–1161.

60. Panigrahi B, Martin KA, Li Y, Graves AR, Vollmer A, Olson L, et al. Dopamine is required for the neural representation and control of movement vigor. Cell. 2015;162(6):1418–1430.

61. Pavlides A, Hogan SJ, Bogacz R. Computational models describing possible mechanisms for generation of excessive beta oscillations in Parkinson's disease. PLoS computational biology. 2015;11(12):e1004609.

62. Tachibana Y, Iwamuro H, Kita H, Takada M, Nambu A. Subthalamo-pallidal interactions underlying parkinsonian neuronal oscillations in the primate basal ganglia. European Journal of Neuroscience. 2011;34(9):1470–1484.

63. Nambu A, Tachibana Y. Mechanism of parkinsonian neuronal oscillations in the primate basal ganglia: some considerations based on our recent work. Frontiers in systems neuroscience. 2014;8:74.

64. Pessiglione M, Guehl D, Rolland AS, François C, Hirsch EC, Féger J, et al. Thalamic neuronal activity in dopamine-depleted primates: evidence for a loss of functional segregation within basal ganglia circuits. Journal of Neuroscience. 2005;25(6):1523–1531.

65. de Lafuente V, Jazayeri M, Shadlen MN. Representation of accumulating evidence for a decision in two parietal areas. Journal of Neuroscience. 2015;35(10):4306–4318.
