# Supplementary material for: CBGTPy: An extensible cortico-basal ganglia-thalamic framework for modeling biological decision making
Source: PLoS One. 2025 Jan 14;20(1):e0310367. doi: 10.1371/journal.pone.0310367 (PMC11731724; doi:10.1371/journal.pone.0310367)
Supplement: S2 Appendix — (PDF) [file pone.0310367.s002.pdf]

S2 Appendix Dopamine-dependent plasticity of corticostriatal synaptic weights

Synaptic plasticity in CBGTPy is implemented using a dopamine-dependent plasticity rule, in which the synaptic updates are governed solely by local factors, without requiring individual neurons to access information about the global system state. This rule is an adaptation of the plasticity mechanism presented in [1].

At each corticostriatal AMPA synapse, the model tracks three key values: eligibility $E(t)$, weight $w(t)$, and conductance $g_x(t)$. The conductance is associated with the synaptic current. How much the conductance grows with each pre-synaptic spike is determined by the weight. The weight is the plastic element in the system, which changes over time depending on the time courses of eligibility and dopamine release.

At a computational level, $E(t)$, which represents a synapse's eligibility to undergo weight modification, depends on the relative spike times of the pre- and post-synaptic neurons involved in the synapse. To compute this quantity, we first define the variables $A_{PRE}(t)$ and $A_{POST}(t)$, which serve as instantaneous estimates of the recent levels of pre- and post-synaptic spiking, respectively. Each time a spike occurs in the pre- or post-synaptic cell, these values are increased by a fixed amount (Δ_{PRE} and Δ_{POST}, respectively), and between spikes, they decay exponentially with a time decay constant τ_{PRE} and τ_{POST}, respectively. That is,

$$\frac{dA_{PRE}}{dt} = \frac{1}{\tau_{PRE}} \left(\Delta_{PRE} X_{PRE}\left(t\right) - A_{PRE}(t) \right),$$
$$\frac{dA_{POST}}{dt} = \frac{1}{\tau_{POST}} \left(\Delta_{POST} X_{POST}\left(t\right) - A_{POST}(t) \right)$$

where $X_{PRE}(t)$ and $X_{POST}(t)$ are sums of Dirac delta functions representing the spike trains of the two neurons. That is,

$$X_{PRE} = \sum_{t_s \in \mathcal{X}_{Cx}} \delta(t - t_s), \qquad X_{POST} = \sum_{t_s \in \mathcal{X}_{SPN}} \delta(t - t_s),$$

where t_s is the spike onset, $\mathcal{X}_{Cx}$ is the set of all cortical neurons projecting to the postsynaptic neuron of interest, and $\mathcal{X}_{SPN}$ refers to the identity of that postsynaptic neuron within the striatum.

Eligibility ($E(t)$) changes over time according to

$$\frac{dE}{dt} = \frac{1}{\tau_E} \left(X_{POST}\left(t\right) A_{PRE}(t) - X_{PRE}\left(t\right) A_{POST}(t) - E \right) \tag{1}$$

where τ_E is a time constant. Note that based on equation (1), $E(t)$ tends toward a level that is boosted whenever a post-synaptic spike occurs soon enough after a pre-synaptic spike and is reduced whenever a pre-synaptic spike occurs soon enough after a post-synaptic spike.

The corticostriatal synaptic conductance g_x takes the value of the synaptic weight, $w(t)$, at each pre-synaptic spike time and decays exponentially in-between these spikes:

$$\frac{dg_x}{dt} = \sum_j w(t_j)\delta(t - t_j) - \frac{g_x}{\tau_{AMPA}},$$

where x stands for the specific connection, t_j denotes the time of the $j-$th spike in the cortical presynaptic neuron, $\delta(t)$ is the Dirac delta function, τ_{AMPA} is the decay time constant associated with AMPA synapses, and w itself changes over time based on

dopamine release and the post-synaptic neuron's eligibility. The evolution of w is given by

$$\frac{dw}{dt} = [\alpha_w^j E(t) f(K_{DA})(w_{max}^j - w)]^+ + [\alpha_w^j E(t) f(K_{DA})(w - w_{min}^j)]^-, \qquad (2)$$

where the nomenclature $[\cdot]^+$ ($[\cdot]^-$) represents a function whose output is the value inside the brackets if it is positive (negative) and 0 otherwise. The learning rate is denoted in equation (2) by α_w^j, for $j \in \{dSPN, iSPN\}$, depending on to which of the two populations the post-synaptic neuron belongs. This rate has a positive sign for dSPN neurons and a negative one for iSPN neurons to reproduce the observation that positive feedback signals lead to a strengthening of the eligible direct pathway connections and a weakening of the eligible indirect pathway connections. Furthermore, w_{max}^j and w_{min}^j are upper and lower bounds for the weight w, respectively, for $j \in \{dPSN, iSPN\}$.

In equation (2), the variable K_{DA} represents the level of available dopamine in the network, which is computed from the amount of dopamine released through the effect of the differential equation

$$\frac{dK_{DA}}{dt} = C_{scale} \sum_i (DA_{inc}(t_i) - K_{DA})\delta(t_i) - \frac{K_{DA}}{\tau_{DA}},$$

where $DA_{inc}(t_j)$ the increment of dopamine, relative to a baseline level, that is delivered at time t_j. That is, after a specific decision i is made at time t_j, a reward value $r_i(t_j)$ associated to action i is received, which induces a dopamine increment based on the reward prediction error

$$DA_{inc}(t_j) = r_i(t_j) - Q_i(t_j),$$

where $Q_i(t_j)$ is the expected reward for action i at time t_j. This expected reward obeys the update rule

$$Q(t_{j+1}) = Q_i(t_j) + \alpha_Q(r_i(t_j) - Q_i(t_j)),$$

where $\alpha_Q \in [0, 1]$ is the value learning rate. More precisely, note that to account for the motor sensory response, the reward is delivered to the network at the end of *phase 1*, $300\,ms$ after the decision is made (see Fig 3 in the manuscript); Q and DA_{inc} are updated together at this reward delivery time, and the update of DA_{inc} in turn impacts the evolution of K_{DA}. Finally, the function $f(K_{DA})$ in equation (2) represents the impact that the available dopamine K_{DA} has on plasticity, such that, if the target neuron lies in the dSPN population, then

$$f(K_{DA}) = \begin{cases} -\gamma, & \text{if } K_{DA} < -\mu, \\ \frac{\gamma}{\mu} K_{DA}, & \text{if } K_{DA} \geq -\mu, \end{cases}$$

while if the target neuron lies in the iSPN population, then

$$f(K_{DA}) = \begin{cases} \varepsilon \frac{\gamma}{\mu} K_{DA}, & \text{if } K_{DA} < \mu, \\ \varepsilon\gamma, & \text{if } K_{DA} \geq \mu. \end{cases}$$

for fixed, positive scaling parameters γ, μ. Parameters values used for the plasticity implementation can be found in Table S2_1

To achieve effective learning, it is critical to address the credit assignment problem of ensuring that the pathways promoting the choice of selected action are the ones that are reinforced by the reward following that action. To achieve this alignment, we introduce a sustained activation signal to the action channel associated with the selected action throughout *phase 1*, based on the patterns of sustained activity that

Parameter	Value
δ_{PRE}	0.8
δ_{POST}	0.04
τ_{PRE}	$15\,ms$
τ_{POST}	$6\,ms$
τ_E	$100\,ms$
α_w^{dSPN}	39.5
α_w^{iSPN}	-38.2
w_{max}^{dSPN}	0.055
w_{max}^{iSPN}	0.035
w_{min}^{dSPN}	0.001
w_{min}^{iSPN}	0.001
ε	0.3
γ	3.0
μ	0.5
C_{scale}	85
τ_{DA}	$2.0\,ms$
α_Q	0.6

Table S2_1. Parameters used for the plasticity implementation.

have been observed in motor planning tasks [2]. Specifically, during this phase, the internal gain of cortical stimulation is altered so that the cortical population corresponding to the selected action maintains elevated activity (at 70% of its firing rate from the end of *phase 0*), while cortical populations corresponding to other actions return to baseline firing now that those actions are no longer under consideration. The localized, sustained cortical activation ensures that the downstream striatal neurons in the appropriate action channel have high eligibility [3].

Taken together, the alteration of the direct-indirect pathway balance increases the tendency of the network to select the rewarded action, giving rise to learning. By using a realistic plasticity rule to produce learning, CBGTPy will enable users to investigate the interplay between the dopaminergic system and basal ganglia dynamics in a way that would be impossible with a less physiologically-accurate learning rule.

References

1. Vich C, Dunovan K, Verstynen T, Rubin J. Corticostriatal synaptic weight evolution in a two-alternative forced choice task: a computational study. Communications in Nonlinear Science and Numerical Simulation. 2020;82:105048.

2. Cisek P, Kalaska JF. Neural Correlates of Reaching Decisions in Dorsal Premotor Cortex: Specification of Multiple Direction Choices and Final Selection of Action. Neuron. 2005;45(5):801–814. doi:10.1016/j.neuron.2005.01.027.

3. Rubin JE, Vich C, Clapp M, Noneman K, Verstynen T. The credit assignment problem in cortico-basal ganglia-thalamic networks: A review, a problem and a possible solution. European Journal of Neuroscience. 2021;53(7):2234–2253.
