# Supplementary material for: CBGTPy: An extensible cortico-basal ganglia-thalamic framework for modeling biological decision making
Source: PLoS One. 2025 Jan 14;20(1):e0310367. doi: 10.1371/journal.pone.0310367 (PMC11731724; doi:10.1371/journal.pone.0310367)
Supplement: S3 Appendix — (PDF) [file pone.0310367.s003.pdf]

S3 Appendix CBGTPy installation and dependencies

The CBGTPy codebase is written in Python 3.8. If the user is using Python version < 3, e.g. 2.7, some of the dependent libraries may not work. Further details about the installation procedure can be found on our github repository.
