# Supplementary material for: CBGTPy: An extensible cortico-basal ganglia-thalamic framework for modeling biological decision making
Source: PLoS One. 2025 Jan 14;20(1):e0310367. doi: 10.1371/journal.pone.0310367 (PMC11731724; doi:10.1371/journal.pone.0310367)
Supplement: S4 Appendix — (PDF) [file pone.0310367.s004.pdf]

S4 Appendix List of files

Here we provide the list of the files that are found on our Github repository and that make up the network, including a short reference to what is implemented in each of them. We distinguish between different sets of files: some are common and used regardless of the type of experiment performed. The remainder have separate versions specific to each experiment type, either the n-choice experiment or the stop-signal task, enabling easier swapping between alternative configurations.

The common files are:

- agentmatrixinit.py: builds the CBGT network.
- backend.py: functions for handling pipeline modules, also connects to the Ray server.
- frontendhelpers.py: deals with the environment variable passed.
- generateepochs.py: where rewards and changepoints are defined; rewards are probabilistic and delivered according to which action has been chosen.
- pipeline_creation.py: creates all modules constituting the pipeline.
- plotting_functions.py: implementation of functions useful for data visualization.
- plotting_helper_functions.py: implementation of functions useful for extracting relevant data.
- postprocessing_helpers.py: contains code to extract the data frames for recorded variables.
- qvalues.py: sets up and updates the parameters for the Q-learning algorithm on every trial.
- setup.py: cythonizes the corresponding core simulator code in agent_timestep.pyx.
- tracetype.py: defines wrapper classes that can pair numeric values with metadata.
- generate_opt_dataframe.py: reads in all optogenetic signal-related parameters and generates a data frame.

The files that are used for the simulation of the plasticity experiments are:

- agent_timestep_nchoice.pyx: contains code for simulating the timesteps of the spiking network.
- init_params_nchoice.py: sets neurons' parameters, receptors' parameters, populations' parameters, dopamine-related parameters for dSPNs and iSPNs, and action channels' parameters with either the defaults or values passed as arguments from the notebook.
- interface_nchoice.py: main simulation controller loop, interacts between environment and the CBGT network.
- popconstruct_nchoice.py: sets up connections between populations and corresponding parameters such as the probability of connection, the mean synaptic efficacy, and the parameters associated with synaptic plasticity (**??**).

The files that belong to the stop-signal task experiment are:

- agent_timestep_stopsignal.pyx: contains code for simulating the timesteps of the spiking network.
- generate_stop_dataframe.py: reads in all stop signal-related parameters and generates a data frame.
- init_params_stopsignal.py: sets neurons' parameters, receptors' parameters, populations' parameters, dopamine-related parameters for dSPNs and iSPNs, and action channels' parameters with either the defaults or values passed as arguments from the notebook; this version differs from the one used to perform the plasticity experiment since different populations are considered for the simulation of the two experiments.
- interface_stopsignal.py: main simulation controller loop, interacts between environment and the CBGT network.
- popconstruct_stopsignal.py: sets connections between populations and corresponding parameters such as the probability of connection, the mean synaptic efficacy, and the parameters associated with synaptic plasticity.
