# Supplementary material for: CBGTPy: An extensible cortico-basal ganglia-thalamic framework for modeling biological decision making
Source: PLoS One. 2025 Jan 14;20(1):e0310367. doi: 10.1371/journal.pone.0310367 (PMC11731724; doi:10.1371/journal.pone.0310367)
Supplement: S5 Appendix — (PDF) [file pone.0310367.s005.pdf]

S5 Appendix Network Scaling

The CBGTPy model allows for the simulation of networks with an arbitrary number of action channels, with a default setting of 2 channels. As the number of channels is varied, certain pathways are automatically adjusted to ensure that the overall quantity of synaptic input to each subpopulation remains constant, allowing the neurons to maintain their proper baseline firing rates. To determine which pathways require scaling, the connectivity pattern of each pathway is compared to a set of cases, which are outlined in Figure S5_1. If, for a given pathway, each target subpopulation only receives input from a single channel or from a shared source, no scaling factor is applied. If, however, each target subpopulation receives input from all action channels, then that pathway requires a scaling factor. This factor is calculated as $2/n$, where n is the new number of action channels. When $n > 2$, the scaling factor is used to reduce the connection probability so that the expected number of afferent synapses per neuron remains constant. As a special case, when $n = 1$ and the scaling factor is 2, the weights of the synapses are increased rather than the connection probability, to avoid potentially setting the pathway's connection probability over 100%. For a detailed listing of connections to which a scaling factor is applied, see Table S5_1.

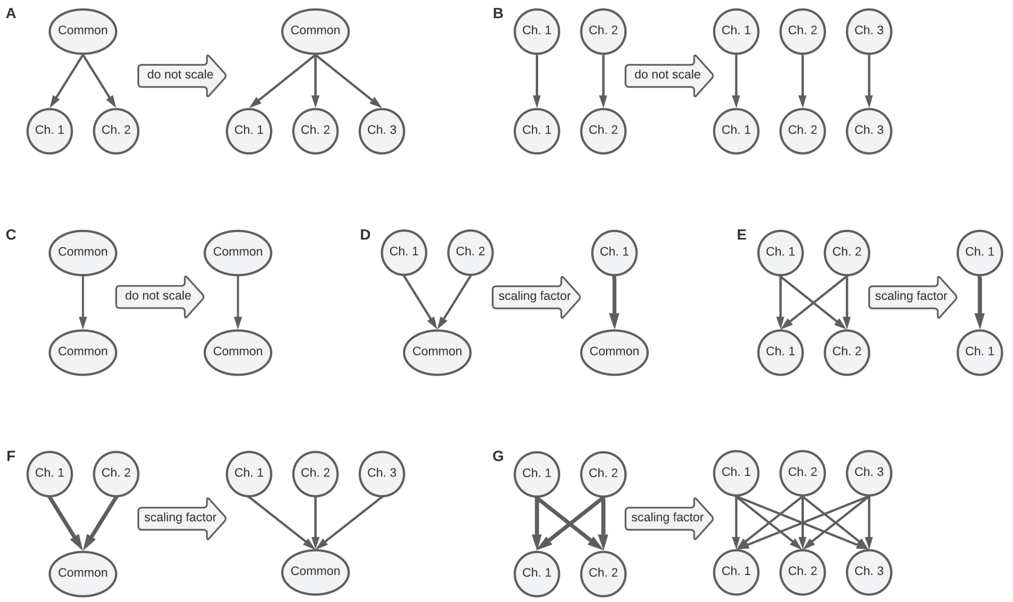

Fig S5_1. Overview of scaling rules. Pathways featuring solely divergent (**A**) or parallel (**B**,**C**) connectivity never have a scaling factor applied. As the number of incoming connections to each target subpopulation remains constant, no scaling of the pathway parameters is needed. When the number of action channels is reduced from 2 to 1, pathways defined by convergent (**D**) or all-to-all (**E**) connectivity have their synaptic weights scaled up by a factor of 2. When the number of action channels is increased above 2, convergent (**F**) and all-to-all (**G**) pathways have their synaptic connection probabilities decreased via the scaling factor.

all pathways network			direct/indirect pathways network		
Connected populations	Receptor type	Scaling rule applied?	Connected populations	Receptor type	Scaling rule applied?
$CxI - CxI$	GABA	no	$CxI - CxI$	GABA	no
$CxI - Cx$	GABA	no	$CxI - Cx$	GABA	no
$Cx - Cx$	AMPA	no	$Cx - Cx$	AMPA	no
	NMDA	no		NMDA	no
$Cx - CxI$	AMPA	yes	$Cx - CxI$	AMPA	yes
	NMDA	yes		NMDA	yes
$Cx - dSPN$	AMPA	no	$Cx - dSPN$	AMPA	no
	NMDA	no		NMDA	no
$Cx - iSPN$	AMPA	no	$Cx - iSPN$	AMPA	no
	NMDA	no		NMDA	no
$Cx - FSI$	AMPA	yes	$Cx - FSI$	AMPA	yes
$Cx - Th$	AMPA	no	$Cx - Th$	AMPA	no
	NMDA	no		NMDA	no
$dSPN - dSPN$	GABA	no	$dSPN - dSPN$	GABA	no
$dSPN - iSPN$	GABA	no	$dSPN - iSPN$	GABA	no
$dSPN - GPi$	GABA	no	$dSPN - GPi$	GABA	no
$dSPN - GPe_A$	GABA	no			
$iSPN - iSPN$	GABA	no	$iSPN - iSPN$	GABA	no
$iSPN - dSPN$	GABA	no	$iSPN - dSPN$	GABA	no
$iSPN - GPe_A$	GABA	no	$iSPN - GPe$	GABA	no
$iSPN - GPe_P$	GABA	no			
$FSI - FSI$	GABA	no	$FSI - FSI$	GABA	no
$FSI - dSPN$	GABA	no	$FSI - dSPN$	GABA	no
$FSI - iSPN$	GABA	no	$FSI - iSPN$	GABA	no
$GPe_A - GPe_A$	GABA	yes			
$GPe_A - iSPN$	GABA	no			
$GPe_A - dSPN$	GABA	no			
$GPe_A - FSI$	GABA	yes			
$GPe_P - GPe_P$	GABA	yes	$GPe - GPe$	GABA	yes
$GPe_P - GPe_A$	GABA	no			
$GPe_P - FSI$	GABA	yes			
$GPe_P - STN$	GABA	no	$GPe - STN$	GABA	no
$GPe_P - GPi$	GABA	no	$GPe - GPi$	GABA	no
$GPe_P - FSI$	GABA	no			
$STN - GPe_P$	AMPA	no	$STN - GPe$	AMPA	no
	NMDA	no		NMDA	no
$STN - GPe_A$	AMPA	no			
	NMDA	no			
$STN - GPi$	AMPA	yes	$STN - GPi$	AMPA	yes
$GPi - Th$	GABA	no	$GPi - Th$	GABA	no
$Th - dSPN$	AMPA	no	$Th - dSPN$	AMPA	no
$Th - iSPN$	AMPA	no	$Th - iSPN$	AMPA	no
$Th - FSI$	AMPA	yes	$Th - FSI$	AMPA	yes
$Th - Cx$	AMPA	yes	$Th - Cx$	AMPA	yes
$Th - CxI$	AMPA	yes	$Th - CxI$	AMPA	yes

Table S5_1. Scaling rule application per connection. Two blocks of 2 columns each are depicted. The first block applies to the full network containing all pathways, while the second block applies to the reduced network containing only the direct/indirect pathways.
