# Supplementary material for: CBGTPy: An extensible cortico-basal ganglia-thalamic framework for modeling biological decision making
Source: PLoS One. 2025 Jan 14;20(1):e0310367. doi: 10.1371/journal.pone.0310367 (PMC11731724; doi:10.1371/journal.pone.0310367)
Supplement: S1 Table — Here we list all those features that the user can modify and those that cannot. If so, we indicate in which table of the Supplementary information the specific parameters are described. (PDF) [file pone.0310367.s006.pdf]

Feature	Table of reference parameters in the manuscript	Specifications	Possibility of modification by the user
Number of neurons considered in each population	S1 Appendix: Table S1_1		Yes
Neural parameters	S1 Appendix: Table S1_2<br>Suppl. Tables: S2 Table, S3 Table, S4 Table, S5 Table		Yes
CBGT connectivity parameters	S1 Appendix: Table S1_3	Receptors type<br>Conn. probability<br>Conn. strength<br>Conn. presence	No<br>Yes<br>Yes<br>Yes
External current parameters	S1 Appendix: Table S1_5	Receptor<br>Frequency<br>Conn. efficacy<br>Conn. number	No<br>Yes<br>Yes<br>No
Parameters used for the plasticity implementation	S2 Appendix: Table S2_1<br>Suppl. Tables: S6 Table, S7 Table, S8 Table		Yes
Parameters used for the stop signal implementations	Suppl. Tables: S9 Table		Yes
Parameters used for the optogenetic implementations	Suppl. Tables: S10 Table		Yes
Scaling rule application per connection.	S5 Appendix: Table S5_1		No

S1 Table. Relation of all parameters editable by the user. Here we list all those features that the user can modify and those that cannot. If so, we indicate in which table of the Supplementary information the specific parameters are described.
