# Supplementary material for: CBGTPy: An extensible cortico-basal ganglia-thalamic framework for modeling biological decision making
Source: PLoS One. 2025 Jan 14;20(1):e0310367. doi: 10.1371/journal.pone.0310367 (PMC11731724; doi:10.1371/journal.pone.0310367)
Supplement: S2 Table — These parameters can be modified through the data frame params. (PDF) [file pone.0310367.s007.pdf]

Parameter	Definition
N	Number of receptors of the neuron
C	Capacitance in nF
$Taum$	Membrane time constant in ms
$RestPot$	Neuron resting potential in mV
$ResetPot$	Neuron reset potential in mV
$Threshold$	Neuron reset potential in mV
$RestPot\_ca$	Resting potential for calcium ions
$Alpha\_ca$	Amount of increment of [Ca] with each spike discharge
Tau_ca	Time constant of Ca-related conductance
Eff_ca	Calcium efficacy
$tauhm$	Duration of the burst in ms
$tauhp$	Duration of hyperpolarization necessary to recruit a maximal post-inhibitory rebound response in ms
$V\_$	Threshold for bursts activation in mV
V_T	Low-threshold of Ca reversal potential in mV
g_T	Low-threshold of Ca maximal conductance in mS/cm^2
g_adr_max	Maximum value of the conductance
$Vadr\_h$	Potential for g_adr_max
$Vadr\_s$	Slop of g_adr at $Vadr\_h$, defining how sharp the shape of g_ard is
$ADRRevPot$	Reverse potential for ADR
g_k_max	Maximum outward rectifying current
Vk_h	potential for g_k_max
Vk_s	Defines how sharp the shape of g_k is
tau_k_max	Maximum time constant for outward rectifying K current
n_k	Gating variable for outward rectifying K channel
h	Gating variable for the low-threshold Ca current

S2 Table. Neuronal parameters editable by the user. These parameters can be modified through the data frame `params`.
