# Supplementary material for: CBGTPy: An extensible cortico-basal ganglia-thalamic framework for modeling biological decision making
Source: PLoS One. 2025 Jan 14;20(1):e0310367. doi: 10.1371/journal.pone.0310367 (PMC11731724; doi:10.1371/journal.pone.0310367)
Supplement: S3 Table — These parameters can be modified through the dictionary pops, addressing the population of interest. (PDF) [file pone.0310367.s008.pdf]

Parameter	Definition
N	Population-specific number of neurons in the nuclei
C	Capacitance in nF
$Taum$	Membrane time constant in ms
g_T	Ca low-threshold maximal conductance in mS/cm^2

S3 Table. Population-specific neuron parameters changeable by the user. These parameters can be modifiable through the dictionary `pops`, addressing the population of interest.
