# Supplementary material for: CBGTPy: An extensible cortico-basal ganglia-thalamic framework for modeling biological decision making
Source: PLoS One. 2025 Jan 14;20(1):e0310367. doi: 10.1371/journal.pone.0310367 (PMC11731724; doi:10.1371/journal.pone.0310367)
Supplement: S4 Table — These parameters can be modified through the data frame receps. (PDF) [file pone.0310367.s009.pdf]

Parameter	Definition
Tau_AMPA	AMPA time constant in ms
$RevPot\_AMPA$	AMPA reversal potential in mV
Tau_GABA	GABA time constant in ms
$RevPot\_GABA$	GABA reversal potential in mV
Tau_NMDA	NMDA time constant in ms
$RevPot\_NMDA$	NMDA reversal potential in mV
$RevPot\_ChR2$	Channelrhodopsin-2 reversal potential in mV
$RevPot\_NpHR$	Halorhodopsin reversal potential in mV

S4 Table. Synaptic and channel parameters changeable by the user. These parameters can be modified through the data frame `receps`.
