# Supplementary material for: CBGTPy: An extensible cortico-basal ganglia-thalamic framework for modeling biological decision making
Source: PLoS One. 2025 Jan 14;20(1):e0310367. doi: 10.1371/journal.pone.0310367 (PMC11731724; doi:10.1371/journal.pone.0310367)
Supplement: S5 Table — These parameters can be modified through the dictionary base, addressing the population of interest. (PDF) [file pone.0310367.s010.pdf]

Parameter	Definition
$FreqExt\_AMPA$	Baseline input firing rate to AMPA receptors
$MeanExtEff\_AMPA$	AMPA conductance
$MeanExtCon\_AMPA$	average of AMPA connections
$FreqExt\_GABA$	input firing to GABA receptors
$TMeanExtEff\_GABA$	GABA conductance
$MeanExtCon\_GABA$	average of GABA connections

S5 Table. Population-specific baseline parameters modifiable by the user. These parameters can be modified through the dictionary `base`, addressing the population of interest.
