# Supplementary material for: CBGTPy: An extensible cortico-basal ganglia-thalamic framework for modeling biological decision making
Source: PLoS One. 2025 Jan 14;20(1):e0310367. doi: 10.1371/journal.pone.0310367 (PMC11731724; doi:10.1371/journal.pone.0310367)
Supplement: S6 Table — These parameters can be modified through the data frame dpmns. (PDF) [file pone.0310367.s011.pdf]

Parameter	Definition
dpmn_DOP	Time constant of the dopamine trace
dpmn_DAt	Tonic dopamine
dpmn_dPRE	Fixed increment for pre-synaptic spiking (Apre)
dpmn_dPOST	fixed increment for post-synaptic spiking (Apost)
dpmn_tauE	Eligibility trace decay time constant
dpmn_tauPRE	Decay time constant for the pre-synaptic spiking trace (Apre)
dpmn_tauPOST	Decay time constant for the post-synaptic spiking trace (Apost)
dpmn_m	Motivation, that modulates the strength of the dopamine level
dpmn_E	Eligibility trace
dpmn_DAp	Phasic dopamine
dpmn_APRE	Pre-synaptic spiking trace
dpmn_APOST	Post-synaptic spiking trace
dpmn_XPRE	Pre-synaptic spike time indicators
dpmn_XPOST	Post-synaptic spike time indicators
dpmn_fDA_D1	f(DA) value for D1-SPNs
dpmn_fDA_D2	f(DA) value for D2-SPNs
dpmn_x_FDA	threshold for f(DA) function
dpmn_y_FDA	threshold for f(DA) function
dpmn_d2_DA_eps	Scaling factor for dopamine levels of D2-SPNs as compared to D1-SPNs

S6 Table. Dopamine-related parameters editable by the user. These parameters can be modified through the data frame `dpmns`.
