# Supplementary material for: CBGTPy: An extensible cortico-basal ganglia-thalamic framework for modeling biological decision making
Source: PLoS One. 2025 Jan 14;20(1):e0310367. doi: 10.1371/journal.pone.0310367 (PMC11731724; doi:10.1371/journal.pone.0310367)
Supplement: S7 Table — Each of the striatal SPN population, dSPN and iSPN, maintain a copy of this data structure which can be independently modified through the data frames dSPN_params and iSPN_params defined in the configuration dictionary in the notebooks. (PDF) [file pone.0310367.s012.pdf]

Parameter	Value
dpmn_type	1 for dopamine-related variables of dSPNs or 2 for iSPN neurons
dpmn_alphaw	weight increment proportional to the dopamine discharge
dpmn_wmax	upper bound for W

S7 Table. Dopamine-related parameters for corticostriatal projections to dSPN and iSPN neurons. Each of the striatal SPN population, dSPN and iSPN, maintain a copy of this data structure which can be independently modified through the data frames `dSPN_params` and `iSPN_params` defined in the `configuration` dictionary in the notebooks.
