# Supplementary material for: CBGTPy: An extensible cortico-basal ganglia-thalamic framework for modeling biological decision making
Source: PLoS One. 2025 Jan 14;20(1):e0310367. doi: 10.1371/journal.pone.0310367 (PMC11731724; doi:10.1371/journal.pone.0310367)
Supplement: S8 Table — For more details about the plasticity parameters, please refer to S2 Appendix. The parameters without a subscript can be modified using data frame dpmns, whereas the parameters with a subscript dSPN or iSPN can be modified through the data frames dSPN_params and iSPN_params respectively. (PDF) [file pone.0310367.s013.pdf]

Parameter	Value
δ_{PRE}	0.8
δ_{POST}	0.04
τ_{PRE}	$15\,ms$
τ_{POST}	$6\,ms$
τ_E	$100\,ms$
α_w^{dSPN}	39.5
α_w^{iSPN}	-38.2
w_{max}^{dSPN}	0.055
w_{max}^{iSPN}	0.035
w_{min}^{dSPN}	0.001
w_{min}^{iSPN}	0.001
ε	0.3
δ	3.0
μ	0.5
C_{scale}	85
τ_{DA}	$2.0\,ms$
α_Q	0.6

S8 Table. Parameters used for plasticity implementation. For more details about the plasticity parameters, please refer to S2 Appendix. The parameters without a subscript can be modified using data frame `dpmns`, whereas the parameters with a subscript `dSPN` or `iSPN` can be modified through the data frames `dSPN_params` and `iSPN_params` respectively.
