# Supplementary material for: CBGTPy: An extensible cortico-basal ganglia-thalamic framework for modeling biological decision making
Source: PLoS One. 2025 Jan 14;20(1):e0310367. doi: 10.1371/journal.pone.0310367 (PMC11731724; doi:10.1371/journal.pone.0310367)
Supplement: S9 Table — The example values included in the table describe the parameters used to generate Fig 7. (PDF) [file pone.0310367.s014.pdf]

Parameter	Description	Example
Optogenetic signal present	List of boolean variables	[True, True]
Optogenetic signal probability	Proportional of trials to be randomly selected or list of trial numbers per nuclei	[[0],[1]]
Optogenetic signal amplitude	Excitatory or inhibitory conductance	[0.5, -0.5]
Optogenetic signal onset	Onset time in ms	[10., 10.]
Optogenetic signal duration	Duration time in ms or phase of the simulation	["phase 0", 400.]
Optogenetic signal channel	List of channels ("all" or channel name)	["all","all"]
Optogenetic signal population	List of nuclei	["iSPN","dSPN"]

S9 Table. Parameters that can be set for optogenetic stimulation. The example values included in the table describe the parameters used to generate Figure 8.
