# Supplementary material for: CBGTPy: An extensible cortico-basal ganglia-thalamic framework for modeling biological decision making
Source: PLoS One. 2025 Jan 14;20(1):e0310367. doi: 10.1371/journal.pone.0310367 (PMC11731724; doi:10.1371/journal.pone.0310367)
Supplement: S10 Table — The example values included in the table describe the parameters used to generate Fig 8. (PDF) [file pone.0310367.s015.pdf]

Parameter	Description	Example
Stop signal present	List of boolean variables	[True, True]
Stop signal probability	Proportional of trials to be randomly selected or list of trial numbers per nuclei	[1., 1.]
Stop signal amplitude	Excitatory conductance	[0.4, 0.4]
Stop signal onset	Onset time in ms	[70., 70.]
Stop signal duration	Duration time in ms or phase of the simulation	[145., 145.]
Stop signal channel	List of channels (“all” or channel name)	[“all”,“all”]
Stop signal population	List of nuclei	[“STN”,“GPeA”]

S10 Table. Parameters that can be set for stop signal stimulation. The example values included in the table describe the parameters used to generate Figure 7.
