# Supplementary figures and images for: CBGTPy: An extensible cortico-basal ganglia-thalamic framework for modeling biological decision making
Source: PLoS One. 2025 Jan 14;20(1):e0310367. doi: 10.1371/journal.pone.0310367 (PMC11731724; doi:10.1371/journal.pone.0310367)

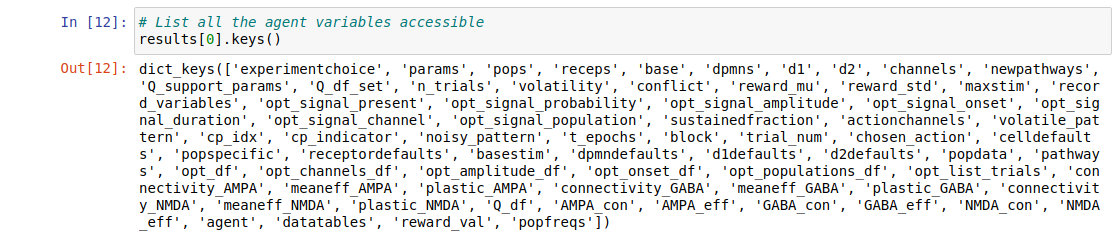

Supplement: S1 Fig — (TIF) [file pone.0310367.s016.tif]

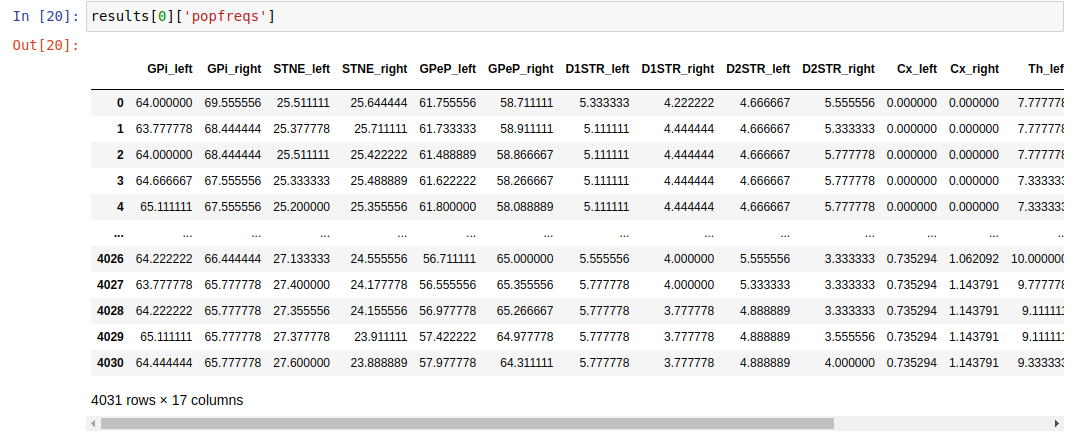

Supplement: S2 Fig — (TIF) [file pone.0310367.s017.tif]

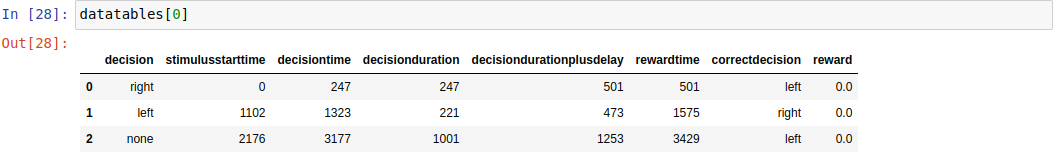

Supplement: S3 Fig — (TIF) [file pone.0310367.s018.tif]

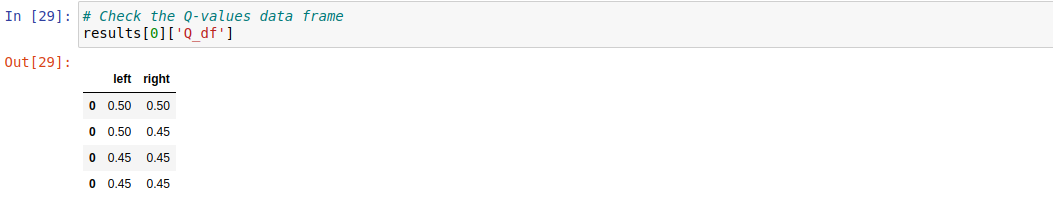

Supplement: S4 Fig — (TIF) [file pone.0310367.s019.tif]
