# Supplementary material for: CBGTPy: An extensible cortico-basal ganglia-thalamic framework for modeling biological decision making
Source: PLoS One. 2025 Jan 14;20(1):e0310367. doi: 10.1371/journal.pone.0310367 (PMC11731724; doi:10.1371/journal.pone.0310367)
Supplement: Attachment — Submitted filename: Reviewers Answers - CBGTPy.pdf [file pone.0310367.s020.pdf]

We thank all reviewers for their comments and helpful suggestions (black text). We have responded in blue text below each of their requests. Changes are highlighted in red on the manuscript.

Reviewer #1: The manuscript "CBGTPy: An extensible cortico-basal ganglia-thalamic framework for modeling biological decision making" presents a biologically relevant, extendable model of the cortico-basal ganglia-thalamo-cortical loop. This model is both tunable to reproduce decision-making tasks and has biological realism to gain insights into the mechanisms of decision making at play.

1. I appreciate versatility of the model. However, it's not clear what features are set by the design and what features can be changed. In particular, is the number of neurons given in the supplements constant or extra neurons or populations can be added? I would appreciate a summary or table that summarizes this.

Reply: We thank the reviewer for appreciating our model and completely agree with the need for clarity on the modifiable features of the network. We have taken your suggestion of adding a table summarizing the list of model features that can be modified. We have included the table below in the manuscript (see Supplementary table S1 Table.) In addition, we have introduced two new tables indicating the parameters that the user can modify with respect to the stop signal (Supplementary Table S9) and the optogenetic (Supplementary Table S10) experiments.

Feature	Table of reference parameter in the manuscript	Specifications	Possibility of modification by the user
Number of neurons considered in each population	S1 Appendix:<br>Table S1_1		Yes
Neural parameters	S1 Appendix:<br>Table S1_2<br>Suppl. Tables:<br>S2 Table, S3 Table,<br>S4 Table, S5 Table		Yes
CBGT connectivity parameters	S1 Appendix:<br>Table S1_3	Receptors type	No
		Conn. probability	Yes
		Conn. strength	Yes
		Conn. presence	Yes
External current parameters	S1 Appendix:<br>Table S1_5	Receptor	No
		Frequency	Yes
		Conn. efficacy	Yes
		Conn. number	No
Parameters used for the plasticity implementation	S2 Appendix:<br>Table S2_1<br>Suppl. Tables:<br>S6 Table, S7 Table,<br>S8 Table		Yes

Parameters used for the stop signal implementations	Suppl. Tables: S9 Table		Yes
Parameters used for the optogenetic implementations	Suppl. Tables: S10 Table		Yes
Scaling rule application per connection.	S5 Appendix: Table S5_1		No

2. Another problem is how the lack of uniqueness of solution may affect their validity. System reconstruction is an inverse problem that has multiple solutions. In large tunable models, fitting procedures may end up with completely unphysiological or otherwise implausible parameter sets. These models fit better if provided not only behavioral, but also electrophysiological data to restrict the solutions. This is not the case for this model as far as I understand. Another way of modeling is much more restrictive models, where the number of neural groups and connections are minimal (or close to that) for a particular task. A couple of papers I can mention here are actually very relevant to the subject of this manuscript, but not cited (https://pubmed.ncbi.nlm.nih.gov/36943842/ https://pubmed.ncbi.nlm.nih.gov/32040519/ https://pubmed.ncbi.nlm.nih.gov/28408878/). However, in the current manuscript, it's somewhere in the middle. Thus, it poses two problems: First is the lack of unique solution I mention before and/or second, the need to calibrate a vast number of parameters that are not tunable (or plastic). These parameters require data for their calibration, which may not be available. I suggest the authors discuss how these issues can be addressed in this model. The two given examples may serve for this purpose as well: how the parameters were calibrated?

Reply: The reviewer is absolutely correct regarding the problem of parameter identification in these sorts of models. We do not include a built in parameter optimization routine because the goals of parameter tuning/fitting depend largely on the nature of the hypothesis being addressed. For example, if one wanted to use CBGTPy to ask a question about cellular level firing rates or other micro/mesoscale dynamics, then the tuning would be on the cell firing rates itself (see https://journals.plos.org/ploscompbiol/article?id=10.1371/journal.pcbi.1010255 for an example of this type of firing). However, someone interested in fits to macroscopic behavioral responses (e.g., reaction times and choices) might want to relax the constraints on cellular-level properties (see https://elifesciences.org/articles/85223 for an example of this type of fit with CBGTPy). In our experience the simultaneous constraints of fine behavioral tuning *and* cellular property fits is a difficult task in its own right and one that, so far as we know, hasn't been demonstrated yet. Therefore, we leave the method of parameter tuning to the user. Our prior work includes examples of genetic algorithms and other approaches we have taken in order to identify working parameter sets.

However, this is likely to be a concern for general readers as well. Therefore, we now include in the Discussion an elaboration of the issue of parameter fitting.

> "One issue that has been left unresolved in our toolbox is the problem of parameter fitting \cite{oyedotun2017simple, abdolrasol2021artificial}. Spiking network models like those used in CBGTPy have an immense number of free parameters. The nature of both the scale and variety of parameters in spiking neural networks makes the fitting problem substantially more complex than that faced by more abstracted neural network models, such as those used in deep learning and modern artificial intelligence \cite{carlson2014efficient, rossant2011fitting}. This is particularly true when the goal is to constrain both the neural and behavioral properties of the network. Models like CBGTPy can be tuned to prioritize matching cellular

level properties observed empirically (for example see \cite{vich2022identifying}) or to emphasize matching task performances to humans or non-human participants (see \cite{bond2023competing}). We view this as a weighted cost function between network dynamics and behavioral performance whose balance depends largely on the goals of the study. To the best of our knowledge, there is no established solution to simultaneously fitting both constraints together in these sorts of networks. Therefore, CBGTPy is designed to be flexible to a wide variety of tuning approaches depending on the goal of the user, rather than constrain to a single fitting method."

However, to help the readers understand the nature of the parameter choices used in our model, we include Table S1_3 in the Appendix, where we have references for the choice of the weights referring to the Arkypallidal pathway simulations and striatal loop as illustration of the way we attempt to constrain the neural dynamics. For the other connections, we redirect the reader to Wei et al 2015, Lo et al 2006, Kumar et al. 2011, Dunovan et al. 2019. We also refer the reader to paper Vich et al. 2022, in which a study is made on the effects of changing these connections and where there is a table with all the references for the choice of the different weights (also added to S1 Appendix as Table S1_5). Then, we have included, in the S1 Appendix, the sentence

"Connections were adjusted to reflect empirical knowledge about local and distal connectivity associated with different populations (see \cite{wei2015, lo2006cortico, kumar2011role, dunovan2019reward} and the specific connection references in Table S1_3), as well as resting and task-related firing patterns (see Table S1_4 and \cite{vich2022identifying}). We note that our connection probabilities are generally high (unlike some experimental work such as \cite{wilson2013active}) due to the fact that we simulate a small number of action channels \cite{klaus2017spatiotemporal}. In addition, the SPN outputs have been scaled to reflect the fact that the SPN population in our model is relatively small."

Population	Baseline FR range (Hz)	Full FR range (Hz)	References.
dSPN	[0, 5]	[0, 35]	[55-58]
iSPN	[0, 5]	[0, 35]	[55-58]
GPe	[40, 90]	[40, 150]	[59-61]
GPi	[40, 90]	[40, 150]	[61]
STN	[10, 35]	[10, 55]	[59-61]
Th	[5, 20]	[5, 85]	[62]
Cx		[0, 100]	[63]
FSI	[5, 40]	[5, 70]	[63]

3. The manuscript is very well written and organized, and I think a minor revision addressing the above comments will suffice.

Reply: We thank the reviewer for their helpful comments. Although minor, they do help to improve the accessibility of the work.

Reviewer #2: Overall, the authors present and describe their CBGTPy software framework, which allows simulating the cortico-basal ganglia-thalamic circuits using Python. The authors describe the software with a lot of detail on how to use it and show several example tasks that CBGTPy supports. Overall, the tool will help researchers conduct modeling/simulation experiments to test their ideas on circuit mechanisms contributing to decision making and learning. The higher level of detail compared to artificial neural networks is a welcome benefit for the computational neuroscience community to explore.

1. Although the tool is described adequately from the naive end-user point of view, there are many details on how the neural network models are constructed that are important for expert computational neuroscientists.
For example, what models are used for individual neurons, integrate-and-fire or conductance-based Hodgkin-Huxley models? Depending on the answer to that question, further details on the modeling framework are needed.

Reply: The individual neurons are modeled as integrate-and-fire-or-burst (IFB) units (Smith et al., J. Neurophysiol., 2000). The choice to use an integrate-and-fire (IF) style framework was made for computational efficiency, relative to models like the Hodgkin-Huxley model, and to allow us to focus on network connectivity and plasticity, rather than details about contributions of specific ion currents, which would be better studied in a smaller-scale model. The choice to use IFB in particular, rather than a regular IF model, was made to endow the network with bursting capabilities like those observed experimentally, and hence to allow the opportunity for future simulations of pathological states (such as those in a Parkinsonian regime, in which CBGT bursting is elevated). We provide this information in Appendix S1.2, as we mention in the Introduction. In the revision process, we have edited the text at the start of Section 2 to include the following statement, along with a referral to the Appendix:

> "Within each region, we model a collection of spiking point neurons, modeled in a variant of the integrate-and-fire framework [Smith et al., 2000] to include the spiking needed for synaptic plasticity while still maintaining computational efficiency."

2. Do the authors use their own integration libraries or are they relying on other platforms, such as the NEURON simulation environemnt or the NetPyNE platform? While I commend the authors if they developed their own platform, of course, that could lead to software fragementation and bugs, many of which may have been solved already with the more established packages. For that reason, I would ask the authors to explain their design decisions/choices more thoroughly, and whether they could have simply interfaced agents/environment software with standard modeling platforms mentioned above.

Reply: This is an excellent point. We perform numerical integration in Python, accelerated via Cython, rather than using platforms such as NEURON or BRIAN. The neuron model we are using in CBGTPy is a relatively simple one, a conductance-based integrate-and-fire model, whereas one of the core strengths of NEURON and NetPyNE are in their ability to simulate multiscale models down to the morphological and biophysical levels. For our project it was necessary to weigh the strengths of those platforms against the additional complexity that would be introduced by them, specifically the additional interfacing required to create the network model and manipulate it throughout the course of simulation. Ultimately we selected to use our own numerical integration, as this enables our agent-environment interface to have very direct and simple control over the network simulation. The flexibility of the agent-environment interface was a core aim of the project, more so than detailed and high-precision simulation of neurons, and the resulting numerical integration code represents only a small fraction of the overall codebase.

We now include a brief description of this in the main text, Section 2, as follows:

> “Numerical integration is performed via custom Cython code, rather than relying on existing frameworks, such as NEURON \cite{Carnevale_Hines_2006}, BRIAN \cite{BRIAN}, or NetPyNE \cite{NetPyNE}, a design choice which simplified the overall software stack. The core strengths of these frameworks are in the simulation of multi-scale or multi-compartment models, whereas one of the strengths of the CBGTPy model is the high level of direct control that can be exerted over the neural parameters throughout the interactions between the network and its environment (see Section 2.1). The integration is performed in a partially-vectorized manner, in which each variable is represented as a list of Numpy arrays, one array per neural population.”

3. In addition to the above questions, I wonder whether the authors could further relate their work to existing, recent models which explored biologically-inspired learning in more complex, simulated environments. I am curious how difficult would it be to extend the CBGTpy framework to allow simulation of arbitrary goal-oriented environments with agents embedded within them? The research community would benefit from being able to interface CBGTPy with software packages such as OpenAI's Gym, where more complex sensory stimuli and decision making would be interesting to explore. Touching on these considerations would broaden the appeal of the software. There are in fact some recent papers that use multi-layer cortical spiking neuronal networks and dopamine-inspired learning to train agents to play video games and perform other behaviors. Comparison of the present work with these models would be interesting and provide further context for the author's work:

- Training spiking neuronal networks to perform motor control using reinforcement and evolutionary learning, 2022
- Training a spiking neuronal network model of visual-motor cortex to play a virtual racket-ball game using reinforcement learning, 2022

Perhaps a naive suggestion, but some of the games mentioned might be cast into the n-choice tasks, since there are limited choices at every time step of the game, and a reward may be produced based on decision. However, games have more complex sensory stimuli, which CBGTPy might not be able to handle, unless it were interfaced with additional neuronal networks that processed the stimuli. If this were not possible, the downside is of course that more naturalistic style behaviors could not be modeled with the CBGTPy framework.

Reply: We thank the reviewer for the important suggestion. Although the aim of developing CBGTPy has been to develop a modeling framework that simulates experimental paradigms frequently used for understanding cognitive concepts (eg. decision making), an extension to include more naturalistic behavior and interfacing with software packages like OpenAI’s Gym is an ambition that we eventually hope to strive towards. However, apart from the obvious implementation challenges, this also includes several conceptual challenges that will require us to make assumptions that are still currently under exploration. For example, the excellent suggestion made by the reviewer to pose some of the games (say virtual racket ball game) as an n-choice task (which would also be the first approach to be used by the authors), is a concept which still is very much under exploration (https://www.ncbi.nlm.nih.gov/pmc/articles/PMC7815426/). This should first be tested systematically using experimental paradigms on simpler network models with less complex parameter fitting constraints (see our response to Reviewer #1 comment #2). Hence, our approach when designing CBGTPy has been to extend its functionality incrementally by starting with simple paradigms that are more popular in non-human research paradigms, where there is ample published evidence on simultaneous behavioral and neural properties. As those experimental paradigms increase in complexity of behavior we can extend the behavioral properties of CBGTPy. For example, we are

currently working on a version of CBGTPy that learns action timing, consistent with studies on continuous vigor control (see https://www.nature.com/articles/nature17639). These sorts of future developments of the network, while exciting, go beyond the goal of the current manuscript.

Nonetheless, we think that this is an important point for the general reader. As such we have added a paragraph to the Discussion highlighting the future potential of this sort of integration with resources like OpenAI Gym, in order to increase the overall extensibility of the system.

> “Because our focus is on matching neural and behavioral constraints based on experimental observations, CBGTPy's environment was designed to emulate the sorts of task paradigms used in systems and cognitive neuroscience research. We purposefully constructed the environment interface to accommodate a wide variety of traditional and current experimental behavioral tasks. These tasks are often simpler in design than the more complex and naturalistic paradigms used in artificial intelligence and, to an increasing degree, cognitive science. Nonetheless, a long-term goal of CBGTPy development is to interface with environments like OpenAI's Gym \cite{brockman2016openai} in order to pvoide not only a mechanistic link towards more naturalistic behavior, but also a framework to test hypotheses about the underlying mechanisms of more dynamic and naturalistic behaviors.

4. in introduction, please provide some references for this statement:
"Although there are a wealth of biologically realistic simulations of cortical and non-cortical pathways in the literature, these are often designed to address very narrow behaviors and lack flexibility for testing predictions across multiple experimental contexts."

Reply: We have now included a reference that reviews all major basal ganglia models and their limitations in the Introduction for the interested readers. Now, the sentence reads as:

> “Although there are a wealth of biologically realistic simulations of cortical and non-cortical pathways that have helped to significantly advance our understanding of BG function, these are often designed to address very narrow behaviors and lack flexibility for testing predictions across multiple experimental contexts \cite{Nelson2014a, Girard2020, Gurney2001, Dunovan2016, vich2020corticostriatal}.”

5. "We note that the inclusion of a biologically-realistic, dopamine-based learning mechanism, in contrast to the error gradient and backpropagation schemes present in standard artificial agents, represents an important feature of the model in CBGTPy."
I agree this is important, and could shed light on how biological systems actually learn.

Reply: We thank the reader for this observation that we, obviously, agree with.

Methods:
6. Is there a reason Ray was chosen rather than the more standard Mpi4py or MPI?

Reply: We chose Ray out of convenience given that it is one of the most widely used platforms for GPU acceleration in machine learning research. We found that it provides a high-level functionality that met our requirements. Since CBGTPy is completely open source, there is no reason why any future user couldn’t rework the code to use Mpi4py or MPI (or any other acceleration tool that comes on the market in the future).

7. Fig. 3 - are there individual spiking neurons modeled or is this a rate-based model? I would like to hear more details of the neuronal circuits and how they are simulated. Is the NEURON or BRIAN simulator used? Or is a custom-written neuronal simulator used?

Reply: Our network model features individual spiking neurons, and the numerical integration is performed using custom code in Cython rather than with NEURON or BRIAN. The network is implemented in a partially-vectorized manner, with each neural parameter represented as a list of 1D Numpy arrays, one array per population. Neural pathways are stored in a multidimensional list of 2D Numpy arrays, each containing synaptic information between one source and one target population. From here, the numerical integration is a rather straightforward translation of the conductance equations into Python code. Although we are sacrificing a certain degree of power by not using an existing platform like BRIAN, the numerical integration code is a small portion of the overall codebase, whose strength lies in the flexibility of the agent-environment interactions.

We have briefly added this information together with the answer to Reviewer #2, Comment #2, in Section 2 of the main text.

8. Q-learning process - I was under the impression that biologically-inspired learning based on dopamine system was used. But then the authors talk about Q-learning, which is a more abstract learning algorithm lacking many biological details/realism. Can the authors clarify which learning algorithm is primarily used in CBGTPy?

Reply: We apologize for introducing confusion about this point. We have added, in Section 2.2 paragraph “Extract relevant data frame” the following text to clarify this point:

> “As part of the model's tuning of dopamine release and associated dopamine-dependent cortico-striatal synaptic plasticity, the model maintains Q-values for each action, updated according to the Q-learning rule…”

9. Section 3.3 - Why are excitatory/inhibitory currents considered optogenetic rather than electrical or other forms of stimulation? It's true that as implemented, they are targeted towards a specific population, but I would not call it "optogenetic", as it bears little similarity to real

optogenetic mechanisms.

Reply: We thank the reviewer for this question. It encouraged us to deliberate further about our implementation of external stimulation. In our revised submission, we have improved our optogenetic implementation to model the action of two opsins, channelrhodopsin-2 and halorhodopsin. Optogenetic input now modulates the conductances of these opsins. The details of the implementation are described in Section 3.3 and S10 Table in the manuscript. To avoid confusion between the implementation of the go, the stop and the optogenetic signals, we have also added briefly how the stop signal is implemented in Section 3.2 and S9 Table. We also updated the figure 8 with the new optogenetic implementation.

Reviewer #3: The authors have introduced an extensible generative package for modeling cortico-basal ganglia-thalamic pathway (GBGT), called CBGTPy, which simulates neuron responses of various experimental tests, mimicking the functioning of the CBGT of mammalian brain. Although the models are simplifications of the real brain circuit, it allowed the authors to address real experimental questions concerning neural dynamics and behavior. They thus the package in a variety of real life applications, simulating environmental and/or external stimuli, showing the related modifications that these stimuli induced in the CBGT dynamics. The authors carried out an excellent job in describing the package and documenting its parameters. Furthermore, they demonstrated the simplicity in the sintax used for defining these models of brain circuit, which allowed theorists and experimentalists to test novel hypothesis without needing to be expert software developer.

Reply: We thank the reviewer for their summary and positive perspective on our work, which we obviously agree with in its entirety.
